# Fishing for data and sorting the catch: assessing the data quality, completeness and fitness for use of data in marine biogeographic databases

**DOI:** 10.1093/database/bau125

**Published:** 2015-01-28

**Authors:** Leen Vandepitte, Samuel Bosch, Lennert Tyberghein, Filip Waumans, Bart Vanhoorne, Francisco Hernandez, Olivier De Clerck, Jan Mees

**Affiliations:** ^1^Flanders Marine Institute (VLIZ), InnovOcean Site, Wandelaarkaai 7, 8400 Oostende, Belgium and ^2^Research Group Phycology, University of Ghent, Krijgslaan 281/S8, 9000 Ghent, Belgium

## Abstract

Being able to assess the quality and level of completeness of data has become indispensable in marine biodiversity research, especially when dealing with large databases that typically compile data from a variety of sources. Very few integrated databases offer quality flags on the level of the individual record, making it hard for users to easily extract the data that are fit for their specific purposes. This article describes the different steps that were developed to analyse the quality and completeness of the distribution records within the European and international Ocean Biogeographic Information Systems (EurOBIS and OBIS). Records are checked on data format, completeness and validity of information, quality and detail of the used taxonomy and geographic indications and whether or not the record is a putative outlier. The corresponding quality control (QC) flags will not only help users with their data selection, they will also help the data management team and the data custodians to identify possible gaps and errors in the submitted data, providing scope to improve data quality. The results of these quality control procedures are as of now available on both the EurOBIS and OBIS databases. Through the Biology portal of the European Marine Observation and Data Network (EMODnet Biology), a subset of EurOBIS records—passing a specific combination of these QC steps—is offered to the users. In the future, EMODnet Biology will offer a wide range of filter options through its portal, allowing users to make specific selections themselves. Through LifeWatch, users can already upload their own data and check them against a selection of the here described quality control procedures.

**Database URL**: www.eurobis.org (www.iobis.org; www.emodnet-biology.eu/)

## Introduction

Progress in information technology has resulted in an increasing flood of data and information. Efficiently mining this sea of data and determining the quality of the data and its fitness for use has become a major challenge of many disciplines. Evaluating and documenting the quality of data has already become a standard practice in several scientific disciplines over many years, e.g. in medicine (1–4), remote sensing (5–7) and gene sequencing (8–10). It is however only in the last decade that its importance—in combination with the assessment of the fitness for use—has become evident for biological sciences, more specifically for biodiversity data and data related to species occurrences (11–15).

Biodiversity is inextricably linked with biogeography (16), which is clear from the many papers that contain both biodiversity and biogeography in their titles, abstracts and keywords (e.g. 17–20). And both concepts are not only essential in research hypotheses, but also in the field of conservation, management (16, 21, 22) and modelling (23–25).

When looking at larger patterns—e.g. on a European or global scale—data are mostly aggregated from a variety of sources. For the marine environment, data on all living marine species from different regional data centres and nodes flow towards the international Ocean Biogeographic Information System (OBIS; www.iobis.org), making marine biogeographic data freely available online. A variety of data is captured, going from data collected during research and monitoring campaigns to data from museum collections or data derived from literature. Given this very diverse nature of data, there is a strong need to be able to assess the quality of these data and provide feedback to the data providers. In addition, a system to assess the completeness of the record needed to be developed, offering specific filters to the users to be able to e.g. only query species records where complete abundance information is available.

Assessing the quality of a distribution record has thus become indispensable, as has the ability to give an indication of the completeness of that record, especially in database infrastructures such as e.g. EurOBIS, OBIS and the Global Biodiversity Information Facility (GBIF; www.gbif.org) that provide access to data from a wide range of sources (e.g. 13, 14). Several actions regarding quality control and data cleaning have already been undertaken on regional or group-specific databases such as SpeciesLink (http://splink.cria.org.br) for Brazilian data collections, Fauna Europaea (26) for European land and freshwater animal species, fish collection databases in relation to FishBase (27) and the Atlas of Living Australia (ALA, http://www.ala.org.au/). However, efforts on quality control and fitness for use for marine biogeographic data were not yet globally organized, as is now presented here for OBIS.

An indication of the completeness can help the user in evaluating whether a particular record is useful for their analysis or not. A distribution record without a timestamp can e.g. be used to get insights in the general distribution of a species but will not be useful for temporal analysis. This illustrates that distribution records, although they do not share the same level of completeness, can be used for a multitude of applications, depending on the user’s needs.

Over the last year, quality control (QC) tools have been developed to be able to document both the quality and completeness of each distribution record within EurOBIS. After extensive testing these QC tools have been implemented in OBIS and extended with extra quality control procedures. This article will elaborate on these recently developed automated quality control procedures and their relevance. In addition, we will demonstrate the importance and usability of these procedures with some use cases. The main goal of these QC steps is to provide a measure of fitness for use of marine biogeographic data both for the scientists and data managers, by offering several tools that help assessing the completeness and validity of distribution records. For a general description of the structure and content of the EurOBIS and OBIS database, we respectively refer to (10, 28, 29).

## Data systems

The quality control procedures were originally developed on EurOBIS, to add quality flags to the available data. Because these data are largely limited to European seas—and a number of QC steps only make sense on a global level (e.g. outlier detection)—the exercise was repeated on the OBIS database, with addition of a number of steps related to outlier analyses.

The QC procedures on EurOBIS were developed in two different ways: (1) as an automated process, to be able to assess the quality and completeness of the records already available within the database and (2) as online web services that can be used by potential data providers and researchers to assess the quality and completeness of their own data prior to use or submission. The former allows data managers to provide feedback to data providers and to check whether they can make their data more complete and correct gaps and putative errors. In addition, the results of the QC steps can be used for specific filtering on the data. The latter return a result report, listing all records that do not comply with a certain QC step. Users can immediately adapt their data and rerun the QC procedures online before analysing or submitting the data to EurOBIS.

EurOBIS is one of the many regional nodes within OBIS and is committed to a continuous support of OBIS, translated in serving its distribution data to OBIS. As the QC procedures also run on OBIS, the results of this can provide a valuable feedback to the other involved nodes and will therefore improve the quality and completeness of the online available records. Both the data providers and the separate nodes would benefit from this. From OBIS, data are sent to the Global Biodiversity Information Facility (GBIF), which would thus imply that GBIF could also only offer marine data that comply with a certain quality standard.

## Quality control procedures

The quality control procedures have been developed for two main reasons. First of all, the available tools offer scientists the opportunity to quality check their data, prior to planned analyses or publishing their data through (Eur)OBIS and they help the (Eur)OBIS data management team in assessing the completeness and quality of the data when making them available online. When incomplete or possibly incorrect data are sent to (Eur)OBIS, the data management team can easily communicate with the provider on the possibly incorrect records based on the assigned quality flags. Secondly, the assigned quality flags can (i) help users in selecting data that are fit for their specific use and purpose or (ii) make it possible to filter records that comply with a certain quality standard and send those to other data systems such as e.g. the European Marine Observation and Data Network (EMODnet).

Each distribution record goes through a series of automated quality control steps, each generating a QC flag. Each QC step is a question that has a yes/no (= 1/0) answer and the result is stored as a bit-sequence (2^(^*^x^*^−^^1)^) where *X* represents the number of the QC flag. The results of all these QC steps are added up and stored in a single QC field in the (Eur)OBIS database, generating a unique integer value for each possible combination of positively evaluated QC steps. An overview of all the QC steps and their corresponding bit-sequence is given in Table 1. Given the different structure and scope of EurOBIS and OBIS, a number of QC steps have been specifically developed for either EurOBIS or OBIS. The majority (17) of the QC steps are, however, available for both data systems.

**Table 1.** Overview of all the QC steps in the EurOBIS database, including the unique bit-sequence (2^(^*^x^*^-1)^, with *X* = number of the QC flag) when the QC step is evaluated positively. The second last column lists whether a QC step is also available to the users through the online web services. IQR = Interquartile range; MAD = Median absolute deviation; SSS = Sea surface salinity; SST = Sea surface temperature

QC-number	Category	Question	Bit-sequence, if answer is yes	Available as online data service	Implemented in
2	Taxonomy	Is the taxon name matched to WoRMS?	2	Yes (taxon match)	EurOBIS + OBIS
3	Taxonomy	Is the taxon level lower than family?	4	Yes (taxon match)	EurOBIS + OBIS
4	Geography: lat/lon	Are the latitude/longitude values different from zero?	8	Yes (check OBIS format)	EurOBIS + OBIS
5	Geography: lat/lon	Are the latitude/longitude values within their possible boundaries?	16	Yes (check OBIS format)	EurOBIS + OBIS
6	Geography: lat/lon	Are the coordinates situated in sea or along the coastline (20 km buffer)?	32	Yes (check OBIS format)	EurOBIS + OBIS
9	Geography: lat/lon	Are the coordinates situated in the expected geographic area (compare metadata)?	256	No, but visual check possible through separate data validation service	EurOBIS
18	Geography: depth	Is minimum depth ≤ maximum depth?	131 072	Not yet available	EurOBIS + OBIS
19	Geography: depth	Is the sampling depth possible when compared with GEBCO depth map (incl. margin)?	262 144	No, but depths per lat-lon can be requested through geographic web services	EurOBIS + OBIS
7	Completeness: date/time	Is the sampling year (start/end) completed and valid?	64	Yes (check OBIS format)	EurOBIS + OBIS
11	Completeness: date/time	Is the sampling date (year/month/day; start/end) valid?	1 024	Yes (check OBIS format)	EurOBIS + OBIS
12	Completeness: date/time	If a start and end date are given, is the start before the end?	2 048	Yes (check OBIS format)	EurOBIS + OBIS
13	Completeness: date/time	If a sampling time is given, is this valid and is the time zone completed?	4 096	Not yet available	EurOBIS + OBIS
14	Completeness: presence/abundance/biomass	Is the value of the field ‘ObservedIndividualCount’ empty or > 0?	8 192	Not yet available	EurOBIS + OBIS
15	Completeness: presence/abundance/biomass	Is the value of the field ‘Observedweight’ empty or > 0?	16 384	Not yet available	EurOBIS + OBIS
16	Completeness: presence/abundance/biomass	Is the field ‘SampleSize’ completed if the field ‘ObservedIndividualCount’ is > 0?	32 768	Not yet available	EurOBIS + OBIS
1	(Eur)OBIS data format	Are the required fields from the OBIS Schema completed?	1	Yes (check OBIS format)	EurOBIS + OBIS
10	(Eur)OBIS data format	Is the ‘Basis of Record' documented, and is an existing OBIS code used?	512	Yes (check OBIS format)	EurOBIS + OBIS
17	(Eur)OBIS data format	Is the value of the field ‘Sex’ empty or is an existing OBIS code used?	65 536	Not yet available	EurOBIS + OBIS
21	Outliers:environment	Is the observation within six MADs from the median depth of this taxon?	1 048 576	Not yet available	OBIS
22	Outliers:environment	Is the observation within three IQRs from the first & third quartile depth of this taxon?	2 097 152	Not yet available	OBIS
23	Outliers:environment	Is the observation within six MADs from the median SSS of this taxon?	4 194 304	Not yet available	OBIS
24	Outliers:environment	Is the observation within three IQRs from the first & third quartile SSS of this taxon?	8 388 608	Not yet available	OBIS
25	Outliers:environment	Is the observation within six MADs from the median SST of this taxon?	16 777 216	Not yet available	OBIS
26	Outliers:environment	Is the observation within three IQRs from the first & third quartile SST of this taxon?	33 554 432	Not yet available	OBIS
27	Outliers:geography	Is the observation within six MADs from the distance to the centroid of this taxon?	67 108 864	Not yet available	OBIS
28	Outliers:geography	Is the observation within three IQRs from the first & third quartile distance to the centroid of this taxon?	134 217 728	Not yet available	OBIS
29	Outliers:geography	Is the observation within six MADs from the distance to the centroid of this dataset?	268 435 456	Not yet available	OBIS
30	Outliers:geography	Is the observation within three IQRs from the first & third quartile distance to the centroid of this dataset?	536 870 912	Not yet available	OBIS

The strength of the quality control procedures is that they not only evaluate a dataset as a whole but also look at each record individually, giving a much more detailed view on the quality and completeness of the data and providing more opportunities to users in their data selection as one dataset may contain several useful records, which might have been rejected if the evaluation had been done solely on the dataset level.

1. ***Data format checks***
  Data made available through (Eur)OBIS need to be compliant with the OBIS Schema, used by OBIS. This OBIS Schema has 74 data and information fields, of which 7 are mandatory and 15 are highly recommended. The remaining fields are classified as optional. For a full overview of the OBIS Schema, we refer to the OBIS website (http://www.iobis.org/node/304). A lot of data providers are making use of the Integrated Publishing Toolkit (IPT) developed by GBIF (30) to exchange their data. By doing so, their data follow the Darwin Core format (31) which slightly differs from the OBIS Schema, which is based on an older version of the Darwin Core format. To avoid confusion, the EurOBIS website includes a mapping between the OBIS Schema field names and the currently used Darwin Core field names (http://www.eurobis.org/data_formats).
  The data format check compares the general format of a dataset with the requirements of the OBIS Schema. When any of the required fields is missing or original field names are not correctly mapped to the field names used within OBIS, then these records are negatively evaluated in the QC procedures and are thus in need of an additional check. Fields that are not part of the OBIS Schema can still be shared with EurOBIS—e.g. through the DarwinCore Archive format (32)—but the corresponding data will—at this time—not be shown through the data portal. If the OBIS Schema recommends the use of certain wording or codes—e.g. in the field ‘BasisOfRecord’—this is also checked. The ‘BasisOfRecord’ defines the kind of data: which can be actual observations (*O*), specimen information from museum collections (*S*) or distribution data derived from literature (*L*), which can already provide a first important data filter for the user.
2. ***Assessment of the completeness and validity of information***
  Besides the basic information of a distribution record (what—where—by whom), the OBIS Schema can capture a lot of other species-related information. A number of the quality checks verify the completeness and soundness of different parts of information in a record. This includes traceability information—e.g. institution code and catalogue number—checking how detailed the date information is, verifying that a given date is possible and—if relevant—if the start date is always before the end date and the minimum depth is always smaller than or equal to the maximum depth.
  A number of QC steps make it possible to distinguish between records that can be used as ‘presence-only’ or where actual counts are available. When a count is given, it is checked whether an indication of the sample size is documented, allowing users to re-calculate the given values to a chosen unit. These QC flags give users the opportunity to e.g. only select those distribution records that have complete abundance information available or where the life stage is documented.
3. ***Taxonomic quality control***
  One of the most important quality checks within OBIS and EurOBIS is related to the given taxon names within a dataset. To quality check these names, (Eur)OBIS makes use of the World Register of Marine Species (WoRMS, www.marinespecies.org) (33) as the taxonomic standard. WoRMS is the most authoritative and comprehensive list of names of marine organisms, including information on synonymy. The host institute for WoRMS is the Flanders Marine Institute (VLIZ) in Belgium and the content of WoRMS is updated and validated by a world-wide network of taxonomic experts (33). Only by linking the given taxon names to a widely accepted marine taxonomic standard, such as WoRMS is it possible to rule out spelling variations and link synonyms to their currently accepted names within (Eur)OBIS. A thorough taxonomic standardization allows the grouping of distribution records in a reliable way for further analysis (12).
4. ***Geographic quality control***
  As EurOBIS and OBIS are biogeographic information systems, verifying the geographic content is as important as verifying the taxonomic data. The geographic checks do not only include a 2D check—latitude and longitude—but they also evaluate the third dimension—depth—if documented in the dataset.
  Several checks relate to the latitude–longitude fields within a given dataset (see Table 1). First of all, it is evaluated whether the coordinates are documented and if the provided values are possible, i.e. be different from zero, be expressed as decimal values in the WGS84 format and fall within the valid boundaries (−90 ≤ latitude ≤ +90 and −180 ≤ longitude ≤ +180). Although 0-0 is a marine position in the Gulf of Guinea (Atlantic Ocean), the odds of having sampled at that exact location is relatively small; All 0-0 cases in OBIS so far were referring to unknown positions, which have been auto-filled by zeros. As both data systems are marine, it is verified whether the sampling locations are located in the marine environment, being seas or oceans. Given the fact that they both receive coastal and estuarine datasets, a land mask accommodating for a 20 km buffer from the coastline (http://www.ngdc.noaa.gov/mgg/shorelines/gshhs.html) is taken into account, hence also including most of the estuarine areas. Although some datasets document the coordinate uncertainty or precision, this information has thus far not been taken into account in any of the quality control steps.
  In nearly all cases, a dataset is accompanied by a detailed metadata description, including text information on the geographical range. Within the metadata information system used for EurOBIS, this geographical range information is coupled to Marine Regions (www.marineregions.org), a standard list of marine geo-referenced place names and areas (34). Based on the available information and shape files within Marine Regions, a comparison is made between the location of the sampling points and the general geographical coverage mentioned in the metadata. If this does not correspond, the relevant sampling locations are flagged as possibly incorrect. When no metadata is available, this check cannot be performed and the record is evaluated as being correct. This check is not yet available on the OBIS database.
  Within the marine environment, the relevance of information on sampling depth cannot be underestimated. Based on depth, it is possible to distinguish between e.g. planktonic and benthic observations or coastal and deep-sea observations. Given its importance, it is valuable to evaluate if the given depth-value related to the species observation is a possible value. This assessment combines the given depth-values with their geographic coordinates and compares this to the General Bathymetric Chart of the Oceans (GEBCO) (35). As not all depth values are registered with the same precision—and fluctuations exist due to e.g. tidal differences—a 100 m margin is taken into account when assigning a quality flag for this check. This margin should also largely account for the fact that the mean depth within a grid can potentially differ from the actual sampling depth, especially in topographically complex areas.
5. ***Outlier analysis***
  Next to the earlier documented QC steps that run both on EurOBIS and OBIS, global geographic and environmental outlier analyses were developed specifically for OBIS, generating 10 more QC flags. These additional outlier analyses use external environmental and geographical (depth) data to assess the credibility of a certain distribution record, when compared with the available distribution records within the checked dataset or within OBIS as a whole. Given the non-normal distribution of the environmental, depth and distance values of the sampling points, the following two robust outlier detection methods are used: (i) the absolute deviation from the median, with a limit at six times the median absolute deviation (MAD) (36, 37) and (ii) an approach based on the Tukey box plot method, with boundaries at three times the interquartile range (IQR) (38). Although a value of three times MAD is already considered as conservative (39), setting the values for the rejection criteria is by definition a subjective decision (37). The values used for the QC flags are based on visual analysis of a subset of the OBIS database and on the fact that a point lying at 6xMAD or 3xIQR from the first or third quartile is considered an extreme outlier (38).
  Six of the outlier checks are related to the environment: these checks compare the locality details of a record with depth, sea surface salinity (SSS) and sea surface temperature (SST) values extracted from the global grids of (1) GEBCO (www.gebco.net; The GEBCO_08 Grid, version 20100927), (2) ETOPO1 Global Relief Model (40) and (3) MARSPEC (Ocean Climate Layers for Marine Spatial Ecology) (41), with the earlier explained decision criteria of 6xMAD and 3xIQR. The depth layers of these three global grids are combined and the average of the two most similar depth values is used to average out inconsistencies between the three bathymetric layers. It needs to be taken into account that due to the used resolution of these depth layers—30 arc-second for GEBCO_08 and MARSPEC and 1 arc-minute for ETOPO1 Global Relief Model—the calculated bathymetric values of the positions can significantly deviate from the values at the exact sampling position due to the resolution of the depth layers. These checks help identifying observations that (possibly) occur outside of their environmental range. The four geographic outlier procedures aim (i) to compare the orthodromic or great-circle distance between the actual sampling locations and the centroid of all sampling locations within a specific dataset and (ii) to compare the distance between the sampling location of a specific species record to the centroid of all the available sampling locations of that particular species within the OBIS database. The quality flag is assigned taking into account the 3xIQR or 6xMAD boundaries. The centroid of a set of sampling points is defined as the point that minimizes the sum of squared geodesic distances between itself and each point in the set and it is calculated from all the initial records except those that have zero coordinates or coordinates that fall out of the valid boundaries for the coordinate reference system WGS84.
  The outlier analyses aim to identify species documented outside of their expected ranges and to reveal possible errors in the taxonomic identification or the assigned latitude and longitude which were not identified through the record-level geographic QC steps, e.g. a missing minus sign to indicate South or West or accidental switching of latitude and longitude values.

## Results

**Figure 1.**
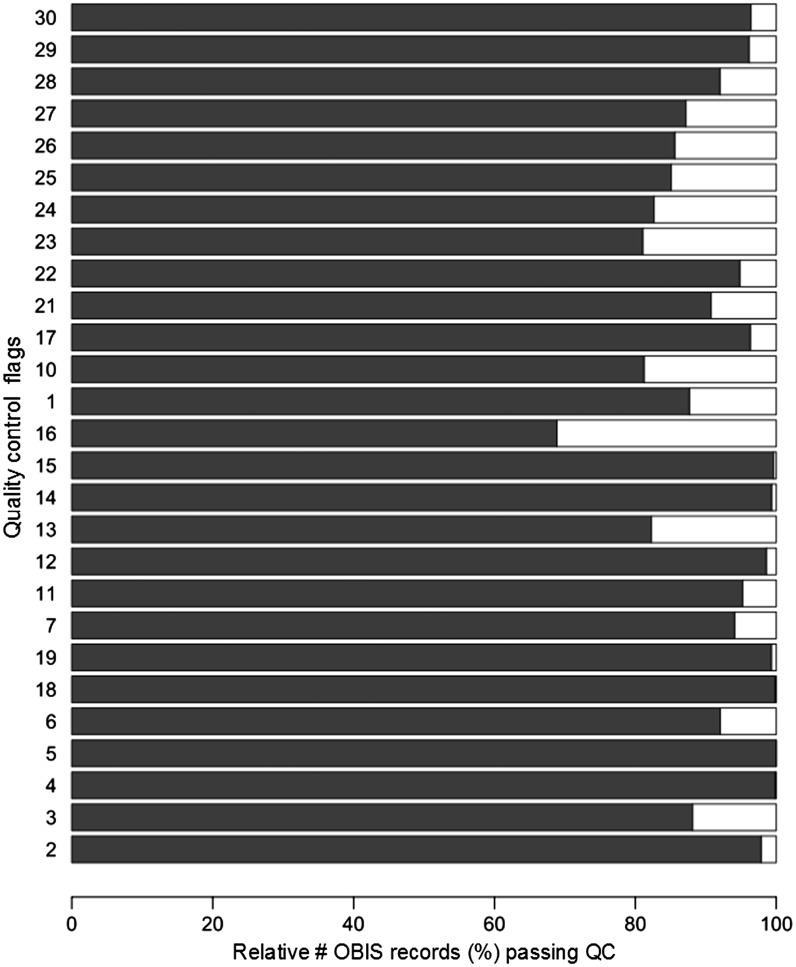
Relative number of records (%) that pass the individual QC steps within the OBIS database. The QC steps are listed in Table 1.

**Figure 2.**
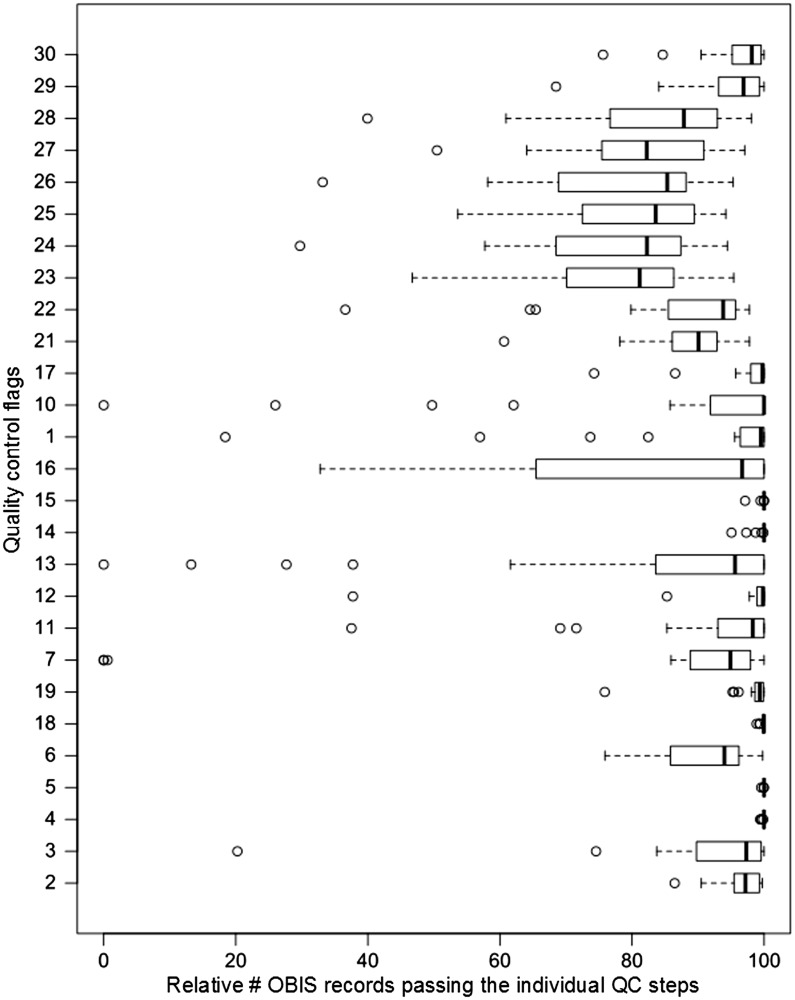
Box and whisker plot per QC step, showing the variability of quality and completeness (in percentage) of the distribution records within the 21 OBIS nodes.

All distribution records within EurOBIS and OBIS have gone through the earlier described quality control steps. Within the OBIS database, at least 60% of the distribution records pass each individual QC step. For some QC steps, >90% of the records pass the enforced criteria (Figure 1). A detailed look shows that the scores of the different OBIS nodes vary greatly (Figure 2), indicating that the results of these QC procedures can provide valuable feedback to the data providers—to double check their data and possibly make corrections and additions—and users, to select the desired data from the system. For an overview of all datasets available within the OBIS database, we refer to http://www.vliz.be/en/imis?module=dataset&dasid=68.

The results show that—on average—85% of distribution records in OBIS and its respective nodes can be used for species or genus specific analyses (Figure 1). All nodes—and thus implicitly OBIS—seem to struggle with capturing the corresponding time zone of the given time at which the data were collected (QC13), which is valuable information when collating data from different time zones. Time and the corresponding time zone information is, e.g. highly relevant when comparing data from different regions and analysing the diurnal vertical migration patterns of, e.g. zooplankton species.

When evaluating the records that contain actual counts (the number of observed individuals within each species) within the (Eur)OBIS database, it becomes clear that the most valuable piece of information—an indication of the sample size—is missing for a large number of records (QC16). As most counts are in essence meaningless without a sample size, this QC result shows that still a lot of work needs to be done to be able to use the count information.

Although the results of the individual QC steps can already give a lot of information on the possible usefulness of a record, it becomes even more useful when several QC steps are combined (Table 2). A selection of relevant QC steps can be made on database level, giving an indication of the distribution records within OBIS that comply to these criteria. In biodiversity research, scientists are specifically interested in geo-referenced species and/or genus data. When combining these selection criteria, almost 85% of the records would be fit for this purpose. The more stringent the criteria become, the fewer records will suit the postulated conditions. The number of suitable records diminishes significantly if one wants to make use of counts or abundance information instead of just presence information (QC16), indicating that this information is rather hard to capture and document within large integrated databases, such as e.g. OBIS.

**Table 2.** Overview of the number of records (absolute and relative) that pass specific combinations of QC steps, indicating their fitness for use in analysing research hypotheses. QC2: taxon name matched to the WoRMS; QC3: taxon level more detailed than family; QC4: coordinates different from zero; QC5: coordinates within possible boundaries; QC6: coordinates in sea or within 20 km coastline buffer; QC7: sampling year available and valid; QC16: count available, in combination with sample size information

Combined QC steps	Positively evaluated OBIS records (#)	Positively evaluated OBIS records (%)
2-3-4-5	34 991 925	86.05
2-3-4-5-6	32 216 817	79.22
2-3-4-5-7	32 849 480	80.78
2-3-4-5-6-7	30 311 653	74.54
2-3-4-5-16	23 315 398	57.33
2-3-4-5-6-16	19 189 668	47.19
2-3-4-5-6-7-16	19 189 668	47.19

**Figure 3.**
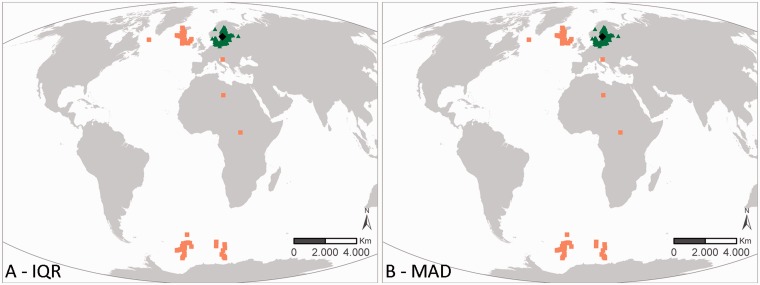
Results of the geographic outlier analysis on the dataset ‘ICES Biological Community’. The left figure (A) represents the IQR approach, the right figure (B) represents the MAD approach. Black diamonds indicate the centroid of the investigated data, green triangles have been evaluated as OK, orange squares have been evaluated as possible outliers.

**Figure 4.**
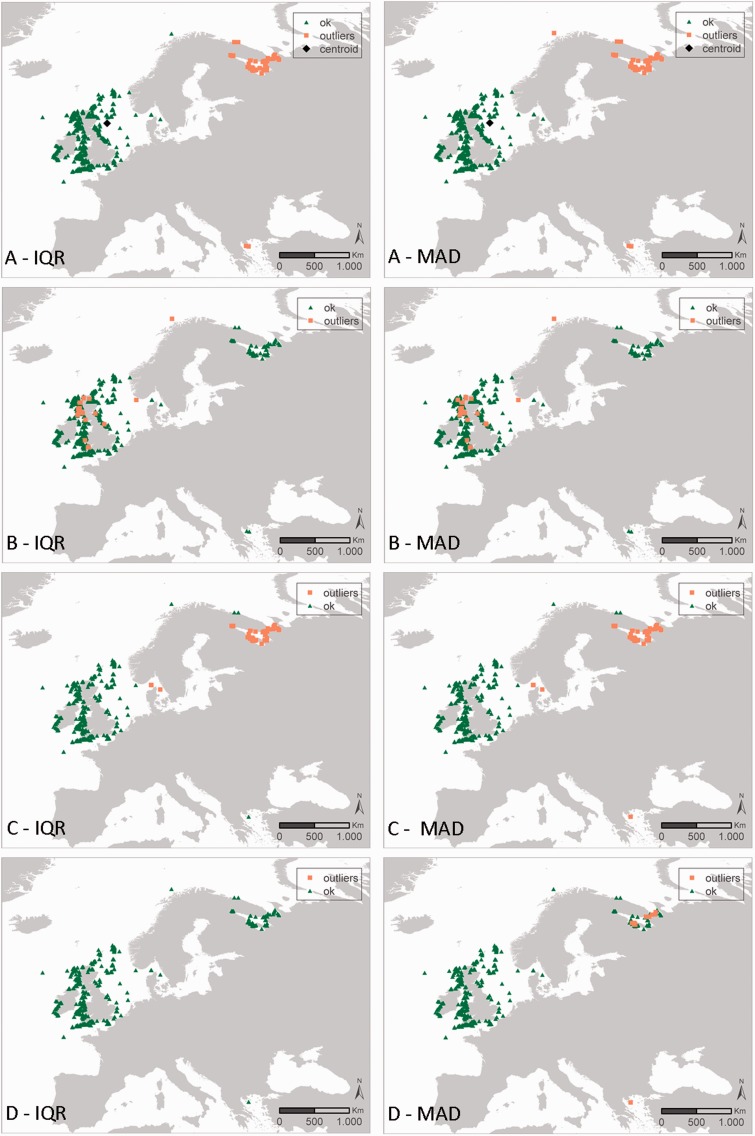
Results of the geographic and environmental outlier analysis of the species *Verruca stroemia* (Crustacea, Cirripedia). The left column represents the IQR approach, the right column represents the MAD approach. The different outlier analyses are A: geography, B: bathymetry, C: Sea Surface Salinity (SSS) SSS and D: Sea Surface Temperature (SST) SST. Black diamonds indicate the centroid of the investigated data (only for the geographic outlier analysis), green triangles have been evaluated as OK, orange squares have been evaluated as possible outliers.

Two different approaches are used within the outlier analyses: the IQR and the MAD methodology. These two have been selected as they are widely used in outlier analyses. In general, the results of both QC procedures are similar. When they differ, the user can combine the results of these QC steps with other QC steps to come to a consensus approach on how to evaluate a specific record. Figures 3 and 4 illustrates that the MAD and IQR approaches can differ, but that these differences are generally relatively small. If a record gets flagged as a possible outlier, some caution is still needed. Figure 3 represents the sampling locations of the dataset ‘International Council for the Exploration of the Sea (ICES) Biological Community’ (42), where the core of the locations is in the Baltic Sea and the other locations are indicated as geographic outliers. After consultation with the data management team at ICES, it became clear that the records in the Antarctic region were the result of a reporting problem in an old format, where positive latitudes were reported as negative. These errors are currently being fixed, and the correct data should soon be available. Possible issues with the Mediterranean, African mainland and Greenland records are not obvious and are still under investigation by ICES.

Figure 4 shows all the distribution records of the Cirriped species *Verruca stroemia* available within OBIS and how they respond to the different geographic and environmental outlier analysis. Appendix S1 gives an overview of the OBIS datasets containing *Verruca stroemia* distribution records. In the ‘distance outlier analysis’, all distribution records along the Norwegian coast, White Sea, Barents Sea and Mediterranean Sea are considered outliers, indicating the species would not occur there. Similar results come from the SSS outlier analysis. Accepting these distribution records as true outliers should be backed up with expert knowledge, as these outliers might not be actual outliers, but e.g. the result of a skewed availability of data within the OBIS database or mis-identifications in the field (see discussion).

## Discussion

The assigned quality flags to each record provide an indication of the ‘fitness for purpose’ of a particular distribution record, helping both the user and the data provider in more objectively assessing the quality and completeness of a record and to draw conclusions from this. The majority of the quality flags do not have the intention to label a record as ‘good’ or ‘bad’, they just give an indication of the completeness and quality, helping the user in his or her decision to make use of a specific record or to reject it.

**Figure 5.**
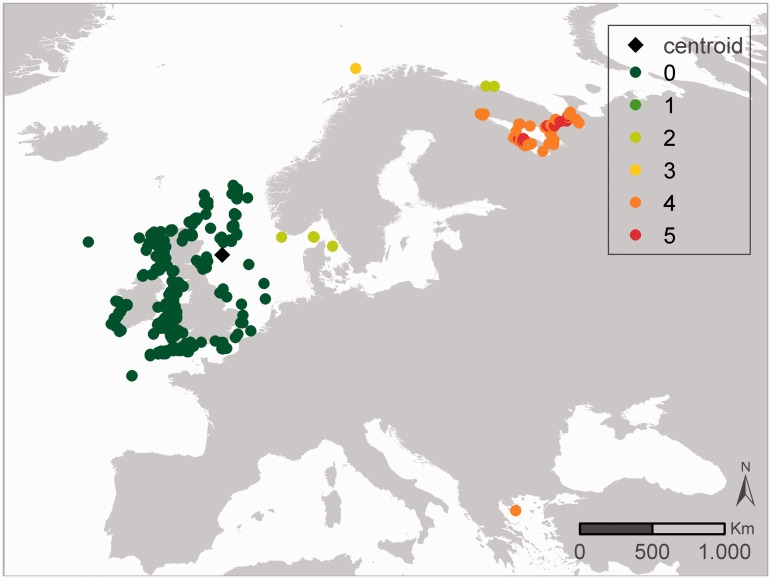
Synthesis map representing the combined results of the outlier analyses of *Verruca stroemia* from Figure 4. The scale represents the number of times a species distribution is seen as an outlier, when combining the eight outlier analyses—geography, bathymetry, Sea Surface Salinity (SSS) and Sea Surface Temperature (SST) SSS and SST according to the IQR and MAD approach—from Figure 4. The black diamond indicates the centroid of the investigated data.

Users need to be aware of the fact that the results of the outlier analyses only provide an indication of the possible outlier character of a distribution record. Records flagged as an outlier are not necessarily true outliers: the distribution of a species can e.g. be unrelated to bathymetry, but highly dependent on temperature or salinity. A single outlier check might thus not clearly identify an outlier (Figure 4), but combining the results of the different outlier checks can indicate with more certainty that a species observation is outside its suspected range (Figure 5). In addition, knowledge on the actual environmental boundaries of species can help in identifying true outliers and filtering of the data. False positives in the species-based outlier detection can be the result of extremely uneven sampling such as for example data from museum collections. Some true positives on the other hand might not be actual outliers, but could be the first observations for a specific species in a geographical area where it was unknown to appear before. The latter could be the case in first observations of alien species that moved to a new area, and these records should be approached with caution. As the dataset-based outlier detection aims to flag possible errors in the geographic coordinates, this will only work well when the dataset is spatially restricted, e.g. if all samples have been taken in the same region such as the North Sea. When wider geographical areas are covered within a dataset, this outlier detection is prone to giving false positives, e.g. due to a biased sampling effort in the available data. This is clearly the case for *Verruca stroemia* (Figure 4): expert and literature consultation have confirmed that the Mediterranean outliers are true outliers, a consequence of misidentification (43). In this case, the providers of these records will be contacted with the expert and literature information. The northern distribution records (Norwegian coast, White Sea, Barents Sea) are, however, validated by literature. In addition, the available depth values also confirmed the species occurs at a depth range from 0 to 548 m (43). Because different outlier analyses are available, it is recommended that users combine the results of these outlier QC checks with each other and with the results of the more basic geography checks. All these combined will make the interpretation of the validity and fitness for use of the records.

***Use-case 1: Quality controlled data available through EMODnet***
As mentioned earlier, the results of the assigned quality control flags can be combined according to the required ‘fitness for use’ for the users, thereby generating the possibility to create specific filters on the available data within EurOBIS and OBIS. EMODnet Biology Portal (http://www.emodnet-biology.eu/) is already making use of such a filter, to offer a specific subset of EurOBIS data to its users. EurOBIS is the data engine behind the Biology Portal of EMODnet, meaning that the data part of the Biology Portal is driven by the EurOBIS data. It was, however, agreed that only those distribution data that comply with QC steps 2-3-4-5—related to taxonomy and basic geography—are offered to the users, thereby making a useful ‘pre-selection’ of the data. Through the portal, users can still see how many distribution records are available in the original dataset and how many have passed the postulated QC steps and are thus available. As of November 2014, 86% or 15.9 million of all the distribution records available in EurOBIS can be consulted through the EMODnet Biology Portal.
***Use-case 2: Selection of QC steps available as web services through LifeWatch***
As of 2012, EurOBIS is part of the central taxonomic backbone of LifeWatch, an E-Science European Infrastructure for Biodiversity and Ecosystem Research which aims at standardizing species data and integrating the distributed biodiversity repositories and operating facilities. Given the importance of standardization, interoperability and being able to assess the quality and completeness of the available data within LifeWatch, a number of the QC steps related to data format, taxonomy and geography that are currently running on the (Eur)OBIS database have been ‘translated’ to interactive, user-friendly web services (http://www.lifewatch.be/data-services). By making use of these freely available data services, data providers, data managers and users are able to make a general assessment of the quality, completeness and fitness for use of their own biogeographic data by simply uploading them to the LifeWatch portal and selecting the QC steps they want to run on their data.

## Future plans and possibilities

Currently, the QC steps are running automatically on both the EurOBIS and OBIS database. A selection of these QC steps is already available online through LifeWatch as a web service. The creation of a customized filter—a combination of several QC steps—is not yet available for the users. Customized filters on EurOBIS will become available through the EMODnet Data Portal, allowing users to define the necessary ‘fitness for use’ of the required data and to refine their search results accordingly. In the future, similar filter options will be developed on the OBIS data. The data download will then also include the corresponding QC flags. The results of the QC procedures currently stored in the database will be used to communicate with the data providers to improve both the quality and completeness of the available data. Specifically the outlier analyses will provide valuable information to improve the correctness of the data. Currently, newly added datasets are thoroughly analysed before they go online, and possible issues are communicated with the data provider immediately. On the other hand, a lot of data have been uploaded to the database before these QC procedures came into place. For these datasets, a communication plan will need to be worked out to discuss the quality control results with the providers, aiming for the highest possible return and improvement of the data quality and completeness. It is important to realize that for some—mostly historical—datasets, the quality status will remain ‘as is’, e.g. when no additional information is available anymore and the original data provider is no longer around to deal with the identified issues.

Within WoRMS, the taxonomic information is currently being expanded with species attributes, such as whether a species belongs to the benthos or plankton, if a species is coastal or deep-sea, what the feeding method, average body size and life span is etc. Once these literature and expert-based traits have been sufficiently documented, they can be incorporated in the QC steps to offer an even higher quality standard to our users. For example, if WoRMS can distinguish between coastal and open ocean species, then this trait can be used as an additional check on the species distribution information: a coastal species (presumably) observed in the open ocean could then be flagged as a possible incorrect record, drawing the attention of the users to this and letting them decide for themselves whether they want to include this record in their download or analysis or not.

## Conclusion

The development and implementation of the described QC steps meets a need to be able to add quality flags to records and to filter out data based on user needs, taking into account the fitness for purpose of the available records. As an array of QC steps is available, users will be able to create specific filters on the data, answering to their specific data needs and requirements.

Although a number of the discussed QC steps are specifically designed to check data meant for EurOBIS and OBIS, a number of other checks can be used widely by the scientific community to quality control their own data before analysis, publication and data sharing. Offering these QC tools as online, user-friendly data services through LifeWatch (www.lifewatch.be) greatly enhances their overall usability for scientists worldwide and meets the needs of the (marine) scientific community to be able to standardize and quality check their data themselves.

Depending on user needs, more QC steps can be added in the future, or existing QC steps could be fine-tuned to better meet their requirements. The mining of a quality controlled, integrated database of different data sources can give insights in previously unexplored matters and offers the possibility to develop new or improved technologies related both to the quality of the data and the outcomes. It is, however, important to realize that the outlier QC results should be approached with due caution. Because the QC steps are automated, a critical analysis of these QC results might be needed to draw the right conclusions on exclusion or inclusion of these records in certain analyses.

## Supplementary data

Supplementary data are available at *Database* Online.

Supplementary Data
