# Supplementary material for: Fishing for data and sorting the catch: assessing the data quality, completeness and fitness for use of data in marine biogeographic databases
Source: Database (Oxford). 2015 Jan 28;2015:bau125. doi: 10.1093/database/bau125 (PMC4309024; doi:10.1093/database/bau125)
Supplement: Supplementary Data [file supp_2015_bau125_index.html]

Fishing for data and sorting the catch: assessing the data quality, completeness and fitness for use of data in marine biogeographic databases — Supplementary Data 

# Fishing for data and sorting the catch: assessing the data quality, completeness and fitness for use of data in marine biogeographic databases

## Supplementary Data

files

**Files in this Data Supplement:**

- Supplementary Data - docx file
