# Supplementary material for: Fishing for data and sorting the catch: assessing the data quality, completeness and fitness for use of data in marine biogeographic databases
Source: Database (Oxford). 2015 Jan 28;2015:bau125. doi: 10.1093/database/bau125 (PMC4309024; doi:10.1093/database/bau125)
Supplement: Supplementary Data [file supp_bau125_Appendix_1.docx]

**Appendix 1 – Overview of the OBIS datasets containing distribution data on *Verruca stroemia***

Allen D., Beckett B., Brophy J., Costello M.J., Emblow C., Maciejewska B., McCrea M., Nash R., Penk M. & Tierney A. Marine species recorded in Ireland during field suveys by EcoServe, Ecological Consultancy Services Ltd. Metadata: <http://www.vliz.be/en/imis?module=dataset&dasid=1947>

Baranova, O.K, T.D. O'Brien, T.P. Boyer and I.V. Smolyar (2009). Plankton data. Chapter 16 in Boyer, T. P., J. I. Antonov , O. K. Baranova, H. E. Garcia, D. R. Johnson, R. A. Locarnini, A. V. Mishonov, T. D. O'Brien, D. Seidov, I. V. Smolyar, M. M. Zweng, 2009. World Ocean Database 2009. S. Levitus, Ed., NOAA Atlas NESDIS 66, U.S. Gov. Printing Office, Wash., D.C., 216 pp., DVDs. Metadata: <http://www.vliz.be/en/imis?module=dataset&dasid=4099>

CEFAS. - UK. Macrobenthos from English waters between 2000-2002. Metadata: <http://www.vliz.be/en/imis?module=dataset&dasid=1681>

Cochrane, S. (2001). Macrobenthos from the Norwegian waters. Akvaplan-niva, Norway. Metadata: <http://www.vliz.be/en/imis?module=dataset&dasid=1856>

Countryside Council for Wales. Marine Nature Conservation Review (MNCR) and associated benthic marine data held and managed by CCW. Countryside Council for Wales, Gwynedd, UK. Metadata: <http://www.vliz.be/en/imis?module=dataset&dasid=657>

Craeymeersh J., P. Kingston, E. Rachor, G. Duineveld, Carlo Heip, Edward Vanden Berghe, 1986: North Sea Benthos Survey. Metadata: <http://www.vliz.be/en/imis?module=dataset&dasid=67>

Dale Rostron. Marine records from Pembrokeshire Marine Species Atlas. Countryside Council for Wales, Gwynedd, UK. Metadata: <http://www.vliz.be/en/imis?module=dataset&dasid=692>

English Nature. Marine Nature Conservation Review (MNCR) and associated benthic marine data held and managed by English Nature. English Nature, Peterborough, UK. Metadata: <http://www.vliz.be/en/imis?module=dataset&dasid=688>

Fisheries Research Service, Marine Laboratory. Macrobenthos samples collected in the Scottish waters in 2001. Metadata: <http://www.vliz.be/en/imis?module=dataset&dasid=1853>

Flanders Marine Institute (VLIZ). Taxonomic Information System for the Belgian coastal area. 10 Aug 2004, Oostende, Belgium. Metadata: <http://www.vliz.be/en/imis?module=dataset&dasid=82>

Hellenic Centre For Marine Research, MedOBIS - Mediterranean Ocean Biogeographic Information System. Hellenic Centre for Marine Research; Institute of Marine Biology and Genetics; Biodiversity and Ecosystem Management Department, Heraklion, Greece. Metadata: <http://www.vliz.be/en/imis?module=dataset&dasid=481>

Mackie, A.S.Y., James, J.W.C., Rees, E.I.S., Darbyshire, T., Philpott, S.L., Mortimer, K., Jenkins, G.O. & Morando, A., 2006. The Outer Bristol Channel Marine Habitat Study. - Studies in Marine Biodiversity and Systematics from the National Museum of Wales. BIOMÔR Reports 4: 249 pp. & Appendix 228 pp. Metadata: <http://www.vliz.be/en/imis?module=dataset&dasid=3068>

Mackie, A.S.Y., P.G. Oliver, E.I.S. Rees, 1991: Biomôr 1 dataset. Benthic data from the Southern Irish Sea from 1989-1991. National Museum and galleries of Wales, Cardiff, UK. Metadata: <http://www.vliz.be/en/imis?module=dataset&dasid=1600>

Marine Conservation Society. Seasearch Marine Surveys. Marine Conservation Society, Ross-on-Wye, UK. Metadata: <http://www.vliz.be/en/imis?module=dataset&dasid=746>

Marine Ecological Surveys Ltd. - UK. Macrobenthos from the eastern English Channel in 1999 and 2001. Metadata: <http://www.vliz.be/en/imis?module=dataset&dasid=1684>

Naumov, A. Benthos of the White Sea. A database. White Sea Biological Station, Zoological Institute RAS. Metadata: <http://www.vliz.be/en/imis?module=dataset&dasid=2769>

Ostler, R. Marine Nature Conservation Review (MNCR) and associated benthic marine data held and managed by JNCC. Joint Nature Conservation Committee, Centre for Ecology and hydrology, Aberdeenshire, UK. Metadata: <http://www.vliz.be/en/imis?module=dataset&dasid=621>

Parr, J. Marine Life Information Network (MarLIN) marine survey data (Professional). Marlin, Collated Marine Life Survey Datasets, Marine Biological Association of the UK, Plymouth, UK. Metadata: <http://www.vliz.be/en/imis?module=dataset&dasid=640>

Picton, B.E., C.S. Emblow, C.C. Morrow, E.M. Sides, P. Tierney, D. McGrath, G. McGeough, M. McCrea,P. Dinneen, J. Falvey, S. Dempsey, J. Dowse, and M. J. Costello, 1999: Marine sites, habitats and species data collected during the BioMar survey of Ireland. Environmental Sciences Unit, Trinity College, Dublin, Ireland. Metadata: <http://www.vliz.be/en/imis?module=dataset&dasid=345>

Rees, H.L., Pendle, M.A., Waldock, R., Limpenny, D.S., Boyd, S.E. A comparison of benthic biodiversity in the North Sea, English Channel and Celtic Seas - Epifauna. Centre for Environment, Fisheries and Aquaculture Science; Burnham Laboratory, 12 Apr 2005, Essex, UK. Metadata: <http://www.vliz.be/en/imis?module=dataset&dasid=505>

Rees, H.L., Pendle, M.A., Waldock, R., Limpenny, D.S., Boyd, S.E. A comparison of benthic biodiversity in the North Sea, English Channel and Celtic Seas - Macroinfauna. Centre for Environment, Fisheries and Aquaculture Science; Burnham Laboratory, 12 Apr 2005, Essex, UK. Metadata: <http://www.vliz.be/en/imis?module=dataset&dasid=3094>

Rigby,P.R., B.Konar, T.Kato, K.Iken, H.Chenelot and Y.Shirayama (2005). NaGISA OBIS Dataset ver.1.. In: NaGISA . 2005. Metadata: <http://www.vliz.be/en/imis?module=dataset&dasid=1983>

Scottish Natural Heritage. Marine species data for Scottish waters held and managed by Scottish Natural Heritage, derived from benthic surveys 1993 to 2012. Scottish Natural Heritage, Edinburgh, UK. Metadata: <http://www.vliz.be/en/imis?module=dataset&dasid=690>

The Danish Biodiversity Information Facility, Marine Benthic Fauna List, Island of Læsø, Denmark. Metadata: <http://www.vliz.be/en/imis?module=dataset&dasid=2038>

The Norwegian Oil Industry Association, 2000: Offshore reference stations, Finnmark. The Norwegian Oil Industry Association (OLF), Akvaplan-niva and Det Norske Veritas, Norway. Metadata: <http://www.vliz.be/en/imis?module=dataset&dasid=998>

The Norwegian Oil Industry Association, 2002: Offshore reference stations, Norwegian/Barents Sea. The Norwegian Oil Industry Association (OLF), Akvaplan-niva and Det Norske Veritas, Norway. Metadata: <http://www.vliz.be/en/imis?module=dataset&dasid=997>

UK National Biodiversity Network, Countryside Council for Wales - Survey of North Wales and Pembrokeshire Tide Influenced Communities. Metadata: <http://www.vliz.be/en/imis?module=dataset&dasid=1883>

UK National Biodiversity Network, Marine Biological Association - DASSH Data Archive Centre Academic surveys. Metadata: <http://www.vliz.be/en/imis?module=dataset&dasid=1890>

UK National Biodiversity Network, Marine Biological Association - DASSH Data Archive Centre expert sighting records. Metadata: <http://www.vliz.be/en/imis?module=dataset&dasid=1885>

UK National Biodiversity Network, Marine Biological Association - DASSH Data Archive Centre volunteer sightings records. Metadata: <http://www.vliz.be/en/imis?module=dataset&dasid=1891>

Wilkinson, S. Marine benthic dataset (version 1) commissioned by UKOOA. Joint nature Conservation Committee, Peterborough, UK. Metadata: <http://www.vliz.be/en/imis?module=dataset&dasid=645>
